# Maternal invalidation and child RSA reactivity to frustration interact to predict teacher-reported aggression among at-risk preschoolers

**DOI:** 10.1017/S0033291722003713

**Published:** 2023-01-05

**Authors:** Amy L. Byrd, Olivia A. Frigoletto, Vera Vine, Salome Vanwoerden, J. Richard Jennings, Maureen Zalewski, Stephanie D. Stepp

**Affiliations:** 1 Department of Psychiatry, University of Pittsburgh, Pittsburgh, PA 15213, USA; 2 Department of Psychology, Queen’s University, Kingston, Ontario, Canada; 3 Department of Psychology, University of Oregon, Eugene, Oregon, USA

**Keywords:** aggression, emotion regulation, emotion socialization, preschool, psychophysiology

## Abstract

**Background.:**

Aggression is a transdiagnostic indicator of risk and represents one of the most common reasons children are referred for mental health treatment. Theory and research highlight the impact of maternal invalidation on child aggression and suggest that its influence may vary based on differences in child physiological reactivity. Moreover, the interaction between these risk factors may be particularly pronounced among children of mothers with emotion regulation (ER) difficulties. The current study examined the independent and interactive effects of maternal invalidation and child physiological reactivity to frustration on teacher-reported aggression in an at-risk sample of preschool children.

**Method.:**

Participants included 77 mothers (*M*_age_ = 33.17 years, s.d. = 4.83; 35% racial/ethnic minority) and their children (*M*_age_ = 42.48 months; s.d. = 3.78; 56% female; 47% racial/ethnic minority). Groups of mothers with and without clinician-rated ER difficulties reported on maternal invalidation, and child respiratory sinus arrhythmia (RSA) was assessed continuously during a frustration task as an indicator of physiological reactivity. Teachers or daycare providers reported on child aggression.

**Results.:**

Results demonstrated positive associations between maternal ER difficulties and both maternal invalidation and child RSA reactivity to frustration. As expected, the interaction between maternal invalidation and child RSA reactivity was significant, such that higher maternal invalidation and greater child RSA reactivity to frustration predicted more aggression in a daycare or preschool setting. Importantly, this effect was demonstrated while controlling for demographic covariates and baseline RSA.

**Conclusions.:**

Findings are in line with diathesis–stress and biosocial models of risk and point to multiple targets for prevention and early intervention.

Aggression is a transdiagnostic indicator that permeates nearly all psychiatric disorders in youth (Kazdin, 2003). It represents one of the most common reasons children are referred for mental health treatment (Kazdin, 2003), and its persistence is linked to severe and intractable trajectories of mental illness (Loeber & Hay, 1997; Ostrov & Houston, 2008; Schaeffer, Petras, Ialongo, Poduska, & Kellam, 2003), as well lower academic achievement, substance use, and incarceration (Huesmann, Dubow, & Boxer, 2009; Kokko, Tremblay, Lacourse, Nagin, & Vitaro, 2006; Tremblay et al., 2004). Theory and research highlight the impact of maternal invalidation on child aggression (Calkins & Hill, 2007; Hajal & Paley, 2020; Morris, Criss, Silk, & Houltberg, 2017; Ramsden & Hubbard, 2002), and suggest that its influence may vary based on differences in child physiological reactivity (Ellis & Boyce, 2008), another known risk factor for aggression (Hubbard et al., 2002; Lorber, 2004; Scarpa & Raine, 1997). Moreover, these effects may be particularly pronounced among children of mothers with emotion regulation (ER) difficulties (Buckholdt, Parra, & Jobe-Shields, 2014; Hajal & Paley, 2020; Rutherford, Wallace, Laurent, & Mayes, 2015), underscoring the importance of examining these factors in at-risk children, before the emergence of serious mental illness. Thus, to refine etiological models and improve prevention efforts, the current study tested the independent and interactive effects of maternal invalidation and child physiological reactivity on aggression among at-risk preschool-aged children.

## Maternal invalidation and risk for aggression

Theories about aggression highlight maternal invalidation as a critical risk factor (Calkins & Hill, 2007; Hajal & Paley, 2020; Morris et al., 2017; Ramsden & Hubbard, 2002). Maternal invalidation refers to communications that emotional experiences or expressions of emotion are inappropriate, invalid, or unwanted (Eisenberg, Cumberland, & Spinrad, 1998). When parents invalidate their child’s emotions, they may respond by dismissing or minimizing emotional experiences (e.g. telling child to change their emotional experience, ‘don’t be angry’), by magnifying or intensifying the emotion (e.g. yelling back, getting angry), and/or or by punishing the expression of emotion (e.g. mocking the emotional experience). To the child, these responses provide immediate feedback about the (un)acceptability of emotions, shaping the way in which emotions are understood, experienced, and expressed in the future (Eisenberg et al., 1998; Morris et al., 2017; Morris, Silk, Steinberg, Myers, & Robinson, 2007). This transactional process may be particularly consequential when socializing negative emotions (Eisenberg, Fabes, & Murphy, 1996), given the centrality of the dysregulation of negative emotion to aggression (Scarpa, Haden, & Tanaka, 2010; Vitaro, Barker, Boivin, Brendgen, & Tremblay, 2006).

Indeed, links between maternal invalidation of negative emotion and aggression have been shown across development, with research documenting associations in childhood (e.g. Brown, Fitzgerald, Shipman, & Schneider, 2007; Chang, Schwartz, Dodge, & McBride-Chang, 2003), and adolescence (e.g. Buckholdt et al., 2014; Byrd, Vine, Frigoletto, Vanwoerden, & Stepp, 2022b). Although theory and research point to the preschool period as particularly sensitive to these maternal influences (Cole, Lougheed, & Ram, 2018), very little work has focused on elucidating risk pathways during this developmental window. The preschool period is characterized by marked neurobiological changesthat underlie notable shifts in emotional and behavioral regulation (Brown & Jernigan, 2012; Garon, Bryson, & Smith, 2008), highlighting the potential importance of clarifying etiological mechanisms early in development, before trajectories of severe mental illness emerge.

## Physiological reactivity and risk for aggression

Research suggests that children with heightened physiological reactivity are also at increased risk for aggression (Lorber, 2004; Scarpa & Raine, 1997). Specifically, children who show extreme reactivity to stress or frustration may be more likely to engage in aggression (Hubbard et al., 2002; Moore et al., 2018; Scarpa et al., 2010), in line with prominent theoretical models of aggression (Berkowitz, 1989, 1993). More recent research has focused on individual variability in peripheral physiology, specifically variation in phasic respiratory sinus arrhythmia (RSA), as an indicator of physiological reactivity and a risk factor for aggression. RSA refers to the amount of variability between heartbeats (i.e. interbeat interval) that rhythmically fluctuates with respiration, and this is typically operationalized as high-frequency (HF) heart rate variability (HRV) using power statistics (Berntson et al., 1997). This non-invasive index is of particular interest, given its unique association with parasympathetic nervous system (PNS) function, specifically vagal control of the sinoatrial node (Berntson, Cacioppo, & Quigley, 1994), and its implicated role in self-regulation (Beauchaine, 2001; Porges, 2007). During rest, tonic PNS activation functions via the vagus nerve to slow heart rate and increase its variability (i.e. higher RSA), which is thought to facilitate emotion and behavior regulation. During stress or frustration, withdrawal of PNS control results in increased heart rate and reduced RSA variability, allowing one to mobilize the metabolic resources needed to optimally respond to environmental demands (i.e. RSA reactivity; Porges, 2007). Consistent with this theoretical perspective, better emotional and behavioral functioning is generally linked to higher tonic RSA and moderate RSA reactivity (specifically RSA withdrawal) to environmental stress or frustration (Graziano & Derefinko, 2013).

While moderate RSA withdrawal is considered an adaptive and necessary response to stress, excessive withdrawal may be problematic, as it is associated with an extreme mobilization of fight or flight responding (Porges, 2007). Although truly dangerous circumstances may require a pronounced physiological reaction to enable necessary behavioral responses, marshaling a pronounced RSA response to everyday frustrations may be less contextually appropriate, and by extension maladaptive. Indeed, excessive RSA reactivity (i.e. greater RSA withdrawal) in response to frustration has been linked to increased risk for aggression (Beauchaine et al., 2019; Byrd et al., 2022a; Fanti et al., 2019). However, most of the work in this area has focused on samples of older children and adolescents, limiting our understanding of these associations early in development. Studies that have examined this association during the preschool period show mixed results, with some linking risk for aggression to excessive RSA withdrawal (e.g. Beauchaine et al., 2013) and others to mild RSA withdrawal or augmentation (e.g. Calkins & Dedmon, 2000). Task differences may be partly responsible for the inconsistent findings (e.g. passive viewing of emotional videos v. engaging in a behavioral frustration task), with some suggestion that more ecologically valid tasks eliciting frustration more reliably produce expected RSA withdrawal (Murray-Close, Holterman, Breslend, & Sullivan, 2017). Additionally, it is possible that mixed results stem from a failure to consider relevant environmental moderators, like maternal invalidation.

## The interaction of maternal invalidation and RSA reactivity and risk for aggression

Existing studies of RSA reactivity and child aggression have rarely examined the potentially moderating impact of maternal invalidation despite theoretical reasons for expecting this effect. Diathesis–stress (Zuckerman, 1999) and biosocial (Beauchaine & Zalewski, 2016; Crowell, Beauchaine, & Linehan, 2009; Linehan, 1993) models suggest that sensitivity to environmental stressors will be more strongly linked with emotional and behavioral problems (e.g. aggression) in environments that are emotionally invalidating. For example, children who experience heightened emotional reactivity in response to stress or frustration *and* experience invalidation of such emotional intensity may have difficultly inhibiting behavioral responses in the context of those emotions, ultimately increasing risk for aggression. Indeed, the interaction between maternal invalidation and physiological reactivity has been associated with emotion and behavior dysregulation (Dixon-Gordon, Marsh, Balda, & McQuade, 2020; McQuade & Breaux, 2017; McQuade, Dixon-Gordon, Breaux, & Babinski, 2021). However, we are aware of only one study that examined this interaction in preschoolers, which demonstrated that mild RSA withdrawal or augmentation and maternal rejection predicted increased aggression (Wagner, Hastings, & Rubin, 2018). This study assessed RSA reactivity to an anger-inducing video and focused on general child-rearing attitudes in a community sample of preschoolers. To better test diathesis–stress and biosocial theories, additional studies are needed that assess RSA reactivity using more externally valid frustration tasks and measure maternal invalidation of negative emotion specifically.

## Maternal ER difficulties as a key risk factor

Not surprisingly, children of mothers with ER difficulties (i.e. experiencing emotional responses as more intense, variable, and prolonged) are at heightened risk for aggression (Rutherford et al., 2015; Zimmer-Gembeck, Rudolph, Kerin, & Bohadana-Brown, 2021). Research points to child physiological reactivity and maternal invalidation as two risk factors that may help to explain this intergenerational transmission (Rueger, Katz, Risser, & Lovejoy, 2011; Zimmer-Gembeck et al., 2021). Children of parents with ER difficulties are more likely to have heightened physiological reactivity (Cao, Powers, Cross, Bradley, & Jovanovic, 2017; Gao, Brown, Neff, Crowell, & Conradt, 2021; Ostlund et al., 2019), placing them at risk for aggression (Lorber, 2004; Scarpa & Raine, 1997). Additionally, because mothers with ER difficulties may find it especially difficult to respond in a validating or supportive manner to their child’s expression of emotion (Buckholdt et al., 2014; Rutherford et al., 2015), they may be more likely to dismiss, magnify, or punish their child’s emotional experience, even if unintentionally (Morelen, Shaffer, & Suveg, 2016; Zimmer-Gembeck et al., 2021). Examining the independent and interactive effects of child physiological reactivity and maternal invalidation among at-risk preschoolers has the potential to enhance etiological models and aid in the identification of modifiable prevention targets that divert youth away from trajectories of severe psychopathology.

## Current study

The current study sought to extend prior work by assessing the independent and interactive effects of child RSA reactivity to an ecologically valid frustration task and maternal invalidation on teacher-reported aggression. Participants were preschool-aged children of mothers with ER difficulties and mothers without ER difficulties. We hypothesized an association between maternal ER difficulties and both child RSA reactivity to frustration and maternal invalidation, and, as outlined in diathesis–stress and biosocial theories (e.g. Crowell et al., 2009; Linehan, 1993; Zuckerman, 1999), that these factors would interact to predict teacher-reported aggression.

## Method

### Sample

We enrolled 90 mothers (*M*_age_ = 33.17 years, s.d. = 4.83; 36.7% racial/ethnic minoritized status^†1^) and their preschool-aged children (*M*_age_ = 42.48 months; s.d.= 3.78; 56% female; 46.7% racial/ethnic minoritized status^2^) in a randomized control trial (RCT) of Dialectical Behavior Therapy (DBT). Mothers were recruited through targeted multimedia and digital messaging programs for mothers of children between 36 and 47 months old (i.e. one advertisement targeting mothers with ER difficulties and one targeting mothers without ER difficulties), via a university-based research recruitment program. Psychiatric treatment clinics were also contacted to aid in the recruitment of mothers with ER difficulties.

At the first point of contact, phone screening interviews were completed to ensure all mothers were a biological parent and had at least 50% physical custody of the target child since birth. To assess for maternal ER difficulties, the 10-item McLean Screening Instrument for Borderline Personality Disorder (MSI-BPD; Zanarini et al., 2003) was completed by all mothers, where scores ≥7 were required for mother with ER difficulties and scores ≤2 were required for mothers without ER difficulties. Given that this was an RCT, mothers with ER difficulties were also asked about current treatment status and those reporting participation in DBT were deemed ineligible. Then, all mothers and their children completed a clinical intake to determine eligibility. This included maternal psychiatric interviews [i.e. Structured Clinical Interview for DSM-5 (SCID-5; First, Williams, Karg, & Spitzer, 2015); Structured Interview for DSM-IV Personality (SIDP-IV; Pfohl, Blum, & Zimmerman, 1995)] to determine maternal ER status. Additionally, neuropsychological testing was administered to mothers and children to obtain an estimate of verbal IQ [i.e. Peabody Picture Vocabulary Test Fourth Edition (Dunn & Dunn, 2003); Expressive Vocabulary Test Second Edition (Williams, 1997)]. All eligible participants demonstrated standard scores of at least 70. Mothers also completed a brief child development screening, the Ages and Stages Questionnaire (ASQ; Squires, Bricker, & Twombly, 2009), designed to detect delays in development across developmental domains, and only children without delays were eligible.

### Procedure

Following the clinical intake, all eligible mothers and their preschoolers were asked to complete four study assessments (baseline, 4-, 8-, and 12-month follow-ups). These assessments included questionnaires assessing emotions and behaviors as well as mother–child interactions (e.g. maternal invalidation). Participants were also asked to complete a series of behavioral tasks, including a child frustration task, during which RSA was continuously recorded. Mothers were also asked to provide contact information for their child’s preschool teacher or daycare provider. Following the assessment, teachers were contacted and asked to complete an online questionnaire assessing emotions and behaviors observed in the preschool/daycare setting. The current study focuses on 77 mother–child dyads recruited between October 2017 and January 2020 who completed an in-person baseline assessment.^3^

### Measures

#### Maternal ER difficulties

Maternal ER status (0 = no ER difficulties; 1 = ER difficulties) was determined during the clinical intake, which included semi-structured diagnostic interviews (i.e. SCID-5, SIDP-IV) administered by trained research personnel. Mothers with ER difficulties met at least three diagnostic criteria for borderline personality disorder, a disorder hallmarked by ER difficulties (Lieb, Zanarini, Schmahl, Linehan, & Bohus, 2004; Linehan, 1993), with one of these three symptoms being affective instability or uncontrolled anger. Mothers with no ER difficulties did not meet diagnostic criteria for any clinical disorder (e.g. depression, anxiety), currently or since conception of their child. Additionally, these mothers showed no evidence of affective instability and uncontrolled anger criteria (i.e. a severity score of 0). Double-coding of approximately 20% of clinical interviews showed strong interrater reliability for maternal ER difficulties (Krippendorff’s *α* = 0.91) and Table S1 details the prevalence of DSM-5 diagnoses in the current sample.

#### Maternal invalidation

Maternal invalidation was assessed using the Coping with Children’s Negative Emotions Scale (CCNES; Fabes, Poulin, Eisenberg, & Madden-Derdich, 2002). The CCNES is a self-report measure consisting of six, 12-item subscales designed to assess parental response to their child’s negative emotions, with three subscales reflecting unsupportive reactions (i.e. minimizing reactions, distress reactions, and punitive reactions). Mothers read several different hypothetical scenarios during which the child feels a negative emotion and following each scenario they rated the likelihood of their responding a certain way on a seven-point Likert-type scale ranging from 1 (*very unlikely*) to 7 (*very likely*). The average of all items from the three unsupportive subscales represented maternal invalidation (range = 1–7). Reliability for this scale was good (*α* = 0.88), and consistent with reliability estimates in similar samples (Meyer, Raikes, Virmani, Waters, & Thompson, 2014; Perry, Calkins, Nelson, Leerkes, & Marcovitch, 2012).

#### Child physiological reactivity to frustration

Child RSA was assessed continuously as an index of parasympathetic function during a 3-minute resting baseline (i.e. watching a Mr. Rogers video) and a 2-minute frustration task. RSA reactivity to frustration was measured using two well-validated frustration tasks: Transparent Locked Box (LAB-TAB; Goldsmith & Rothbart, 1996) and Knotted Sack (Chaplin, Klein, Cole, & Turpyn, 2017).^4^ In each task, the child was allowed to pick their favorite of three toys. The experimenter then placed the chosen toy inside a locked, transparent box or an opaque cloth sack and told the child they could keep the toy once they opened the locked box or knotted sack. Unbeknownst to the children, those in the box condition were then handed an incorrect set of keys, and those in the sack condition were handed an alternate sack that was glued shut. The experimenter explained that they ‘had some work to do’ and turned away from the child as if engrossed in work. The child attempted to open the locked box or knotted sack for 2 minutes.

Electrocardiogram signals were obtained from three disposable Ag/Ag-Cl spot electrodes positioned in a modified lead-II configuration using Mindware BioLab software (MindWare Technologies, Ltd., Gahanna, OH, USA). To estimate RSA, Mindware HRV 3.2.6 software (MindWare) was utilized. The interbeat interval (IBI) series was resampled in equal 250 ms intervals, linearly detrended, and tapered using a Hanning window. Trained scorers independently visually inspected each recorded waveform and manually corrected artifacts based on recommendations set forth by Berntson et al. (1997). Twenty percent of participants were double-scored for reliability (mean Krippendorff’s *α* = 0.89), and any discrepancies were resolved with consensus. HRV was calculated using fast Fourier transformation analysis of the IBI series, and RSA was defined as HRV associated with the log transformed HF respiratory power band (0.24–1.04 Hz range; Shader et al., 2018; West, Shaffer, Wickrama, Han, & Suveg, 2021). Data were available for 95% (*n* = 73) of the sample,^5^ and peak respiration frequency ranged from 0.26 to 0.84. RSA was estimated separately for the resting baseline (*mean* = 6.02; s.d. = 1.38) and for the frustration task (*mean* = 5.40; s.d. = 1.18). To measure within-individual RSA reactivity, a difference score was calculated by subtracting RSA during the resting baseline from RSA during the frustration task. Thus, negative scores reflect RSA withdrawal (reduced RSA during frustration relative to baseline), while positive scores reflect RSA augmentation (increased RSA during conflict relative to baseline). In this sample, mean RSA reactivity was negative (*mean* = −0.62; s.d. = 0.67; *range* = −2.58–0.64), with approximately 85% of preschoolers experiencing RSA withdrawal, and approximately 15% experiencing mild RSA augmentation.

#### Child aggression

To assess aggression, preschool teachers or daycare providers completed the Caregiver-Teacher Report Form (C-TRF; Achenbach & Rescorla, 2001) via the Qualtrics survey system (Qualtrics, Provo, UT). The C-TRF contains 25 items assessing aggression in children who are between 1.5 and 5 years old (e.g. *defiant, physically attacks people, screams a lot*). All items are rated on a three-point Likert scale, ranging from 0 (*not true*) to 2 (*very true or often true*), and are summed to create a total score. Data were available for 83% of the sample (*n* = 64),^6^ and reliability for this scale was excellent (*α* = 0.95).

#### Demographic covariates

Child age, racial/ethnic minoritized status (0 = white; 1 = minoritized status), and child sex (0 = male; 1 = female) were obtained via maternal report. Annual income and family receipt of public assistance were obtained via maternal report (e.g., food stamps, welfare, etc.; 0 = no public assistance; 1 = receipt of public assistance).

### Data analytic strategy

Preliminary analyses were conducted to examine descriptive statistics and bivariate correlations between study variables using IBM SPSS Statistics (Version 26.0). Primary analyses were conducted in MPlus version 8 (Muthén & Muthén, 2017) using full information maximum likelihood with robust standard errors to handle missing data. The independent and interactive effects of maternal invalidation and child RSA reactivity on teacher-reported aggression were estimated simultaneously while controlling for theoretically relevant demographic covariates (i.e. child age, child sex, child minoritized status, family receipt of public assistance), and child baseline RSA. The effects of maternal ER difficulties on maternal invalidation, child RSA reactivity, and teacher-reported aggression were also included in the model. Model fit was evaluated using standard criteria for *χ*^2^, comparative fit index (CFI; Bentler, 1990), and the root mean square error of approximation (RMSEA; Browne & Cudeck, 1993). For CFI, conventional cut-off values of 0.95 or greater indicate good fit (McDonald & Ho, 2002). RMSEA values below 0.05 represent good fit (Kline, 2015; McDonald & Ho, 2002). Standardized effects from the full model are reported. Significant interaction effects were probed and plotted for interpretation using maternal invalidation values ± 1 s.d. above and below the mean. Fraley’s (2018) online utility for parsing the two-way interactions was used to assess simple slopes and regions of significance.

## Results

### Descriptive statistics

Table 1 includes descriptive statistics for demographic and primary study variables and Table 2 shows bivariate correlations. Maternal ER difficulties were associated with higher maternal invalidation, more excessive child RSA withdrawal to frustration, and greater teacher-reported aggression. Maternal invalidation was positively associated with teacher-reported aggression. Child RSA reactivity was negatively associated with teacher-reported aggression, though not significantly.

#### Independent and interactive associations with aggression

**Fig. 1.**
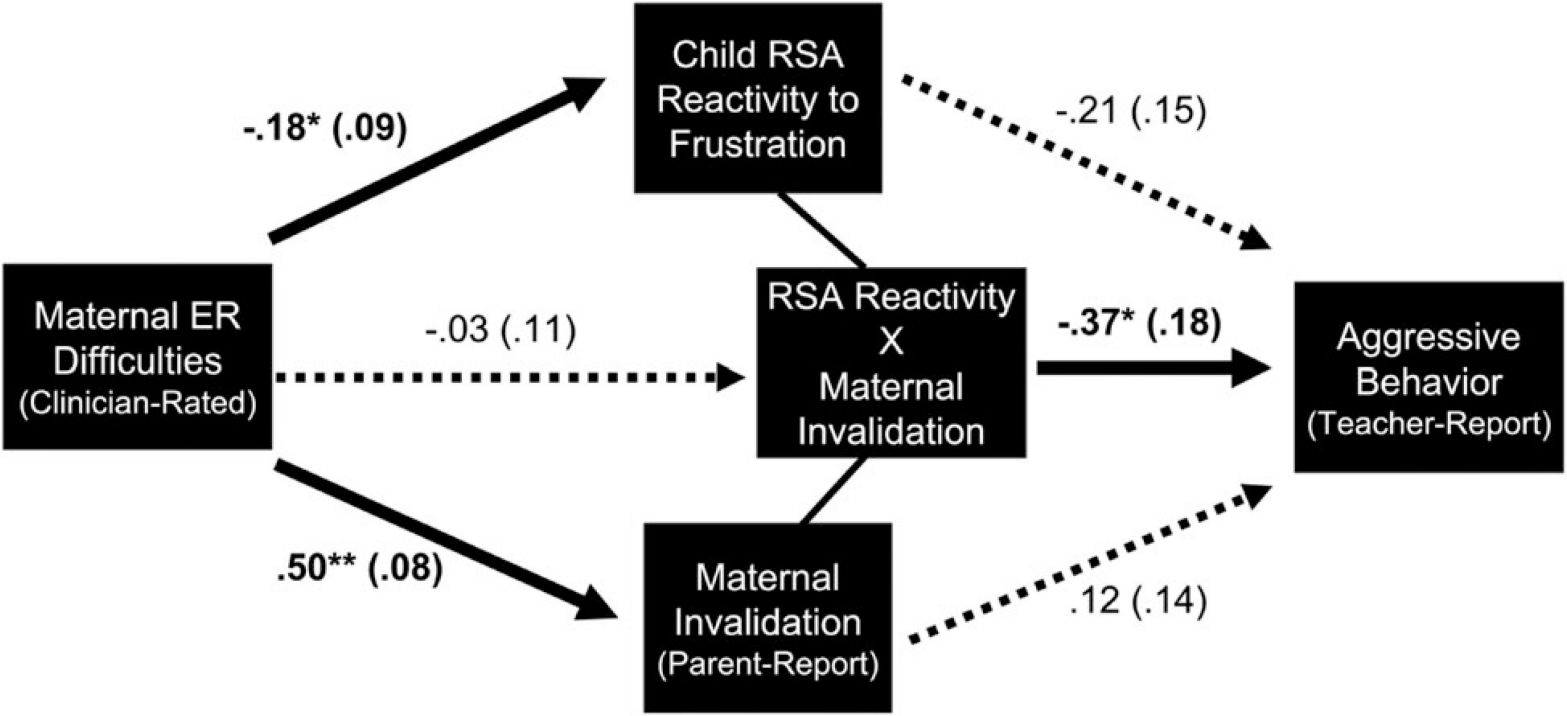
Multivariate model testing independent and interactive paths from maternal ER difficulties, maternal invalidation, and RSA reactivity to frustration to teacher-reported aggression. *Note*. Overall model fit was good [χ^2^(11) = 8.50, p = 0.67; CFI = 1.00; TLI = 1.00; RMSEA < 0.01]. *R*^2^(aggressive behavior) = 0.33. Significant associations are bolded and represent standardized effects after accounting for child age, child sex, child minority status, family receipt of public assistance, and child baseline RSA. The direct path between clinician-rated maternal ER difficulties and teacher-reported aggression was also modeled (though not shown above) and was non-significant [*β* = 0.19 (0.14), *p* = 0.18]. *p < 0.05; **p < 0.01.

**Fig. 2.**
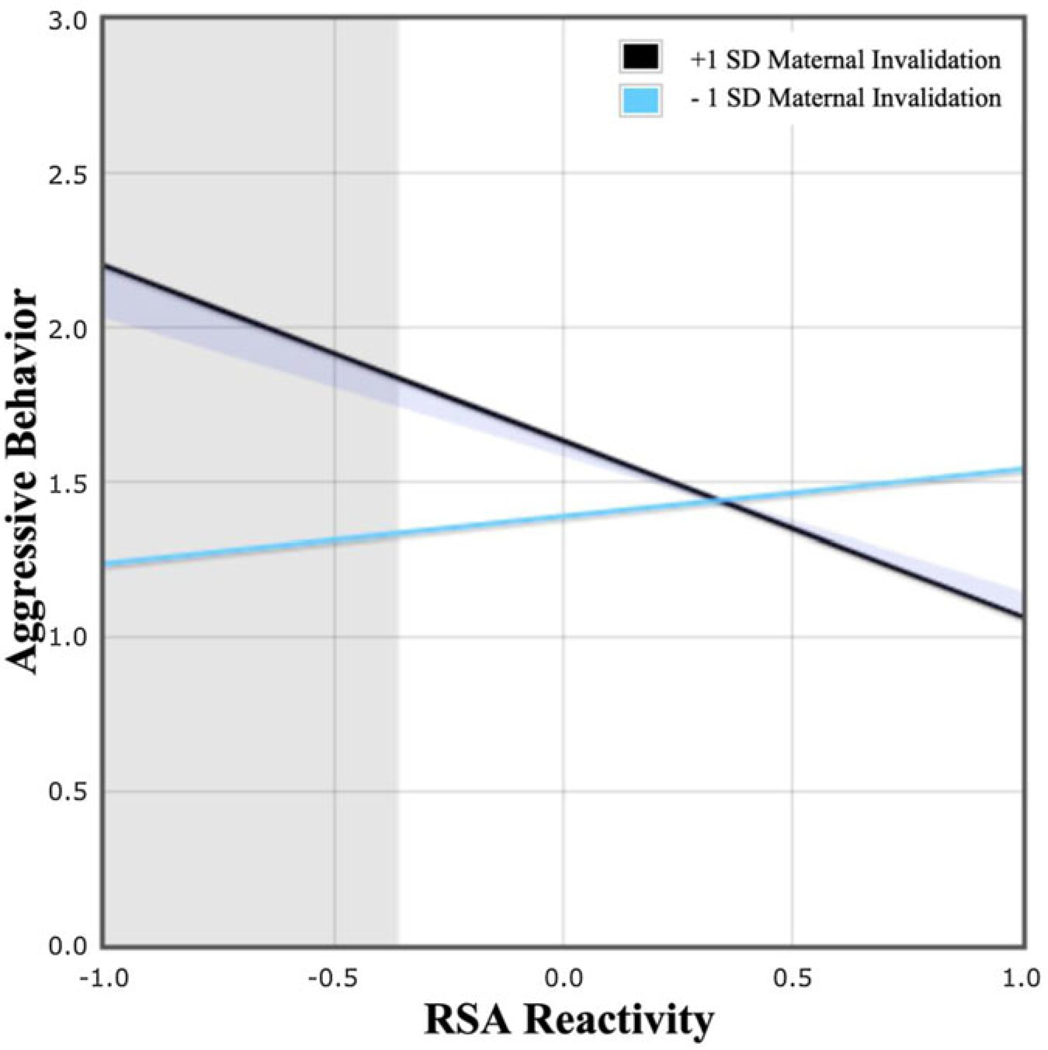
Maternal invalidation moderates the effect of child RSA reactivity to frustration on teacher-reported aggression. Predictors were mean-centered, and effects are shown at ±1 s.d. above and below the mean on maternal invalidation (mean = 2.39; s.d. = 0.67; range = 1.31–4.61), with higher values reflecting more maternal invalidation, and lower values reflecting less maternal invalidation. Simple slope analyses revealed that child RSA reactivity was related to teacher-reported aggression at higher levels of maternal invalidation (+1 s.d.: *b* = − 0.57, *p* = 0.03), but not at lower levels of maternal invalidation (−1 s.d.: *b* = 0.15, *p* = 0.29). The overlapping shaded areas represent the point beyond which child RSA reactivity, specifically RSA withdrawal (< −0.36; mean-centered range = −2.93 to 1.87), predicted teacher-reported aggression for preschoolers with higher maternal invalidation (>0.67; mean-centered range = −1.62 to 3.35).

Figure 1 shows the multivariate model testing the independent and interactive effects of maternal invalidation and child RSA reactivity to frustration on teacher-reported aggression. Maternal ER difficulties were associated with higher levels of maternal invalidation and more excessive RSA withdrawal to frustration. Additionally, the interaction between maternal invalidation and child RSA reactivity was significantly associated with teacher-reported aggression. Post-hoc simple slope analyses showed that RSA reactivity to frustration was related to teacher-reported aggression at high levels of maternal invalidation (+1 s.d.), but not low levels of maternal invalidation (−1 s.d.). Region of significance analyses indicated that higher maternal invalidation was significantly associated with more teacher-reported aggression among children with more extreme RSA withdrawal (Fig. 2).^7^

## Discussion

The current study examined the independent and interactive effects of maternal invalidation and child RSA reactivity to frustration as predictors of teacher-reported aggression in an at-risk sample of preschoolers. As expected, results demonstrated positive associations between clinician-rated maternal ER difficulties and both maternal invalidation and child RSA reactivity to frustration. As hypothesized, there was a significant interaction between maternal invalidation and child RSA reactivity, such that higher levels of maternal invalidation predicted more aggression in a preschool/daycare setting among children who experienced more excessive RSA withdrawal to frustration. Importantly, this effect was demonstrated while simultaneously accounting for the effects of demographic covariates and child baseline RSA. Findings are in line with diathesis–stress and biosocial models of risk and point to multiple targets for prevention and early intervention.

Mothers with ER difficulties reported higher maternal invalidation, and their children had more excessive RSA withdrawal to frustration, echoing previous research demonstrating associations between maternal ER difficulties and maternal invalidation of emotion and child physiological reactivity (Cao et al., 2017; Frigoletto et al., 2022; Gao et al., 2021; Ostlund et al., 2019; Zimmer-Gembeck et al., 2021). Mothers with ER difficulties may feel overwhelmed by their own internal emotional experience when attempting to navigate challenging emotional interactions with their child, making it more difficult to respond effectively (Rutherford et al., 2015). These interactions can be emotionally evocative for any parent, and given that children of mothers with ER difficulties may also have greater physiological reactivity to frustration (Cao et al., 2017; Gao et al., 2021; Ostlund et al., 2019), future work should examine whether this is even *more* challenging for mothers with ER difficulties. Because preschoolers still primarily rely on external influences to aid in regulation (Thompson, 1994), modifying maternal responses to child emotion during this time has the potential to significantly shape child outcomes. Taken together, these findings highlight the importance of characterizing these influences during the preschool period, a sensitive developmental window marked by notable shifts in emotional and behavioral regulation (Brown & Jernigan, 2012; Garon et al., 2008).

Our results demonstrated a significant interaction between maternal invalidation and child RSA reactivity. Specifically, children who experienced RSA withdrawal to frustration and higher maternal invalidation showed more aggression in the preschool or daycare setting. This finding suggests that the impact of maternal invalidation may not be equivalent across all children and demonstrates that the most deleterious effects may be felt by those children with heightened physiological reactivity, in line with the diathesis–stress and biosocial models (e.g. Crowell et al., 2009; Linehan, 1993; Zuckerman, 1999). Together, these factors placed children at higher risk for aggression during preschool, a developmental window typically characterized by a normative decline in aggression (Hartup, 1974; Tremblay et al., 2005), increasing their risk for a wide range of psychopathology later in development (Loeber & Hay, 1997; Ostrov & Houston, 2008; Schaeffer et al., 2003). For children with heightened physiological reactivity, emotional scaffolding and validation may be even more important, as invalidation in the context of heightened physiological reactivity may limit crucial opportunities for youth to learn how to regulate their emotions, and over time, exacerbate emotional distress, increasing risk for aggression. Along these lines, recent meta-analytic work shows that physiological reactivity, and specifically RSA withdrawal, decreases during the first 3 years of life (Wagner, Holochwost, Lynch, Mills-Koonce, & Propper, 2021), with some suggestion that maternal scaffolding and validation may directly support this trajectory (Perry, Dollar, Calkins, & Bell, 2018). Taken together, these findings underscore the sensitivity of this developmental window, when prevention and intervention efforts may be critical and potentially most effective.

### Limitations

Findings from the current study should be considered within the context of several limitations. First, this study focused on an at-risk sample of preschoolers at a single timepoint, limiting the generalizability of findings to community samples and our ability to assess effects over time. Second, while several important covariates (e.g. age, sex, minoritized status) were included in our model, this study was not adequately powered to examine potential moderation. Moreover, minoritized status was dichotomized and utilized in statistical models as a person-centered variable. Race is a non-discrete, socially created construct and reflects the effects of numerous risk factors (e.g., minority stress) for which minoritized status is a proxy (Kaufman & Cooper, 2001; Richeson & Sommers, 2016). Future work is needed to examine the direct impact of these factors on maternal ER difficulties, maternal invalidation, and aggression (Jones & Neblett, 2017).

Additionally, while we assessed invalidation using a well-validated maternal-reported questionnaire (i.e. CCNES), research highlights children’s perception or experience of validation as most predictive (e.g. Byrd et al., 2022b), and future work should utilize alternative assessment methods and informants. Finally, while our assessment of peripheral physiology expands on previous laboratory-based assessments (i.e. passive viewing of emotional stimuli) by utilizing a more ecologically valid frustration task, we acknowledge the importance of assessing task-induced artifacts and the benefits of utilizing standardized methods across studies (Davis, Brooker, & Kahle, 2020). Additionally, although we focused on RSA as an indicator of parasympathetic function and used difference scores to assess reactivity, we understand that other autonomic influences are likely at play (i.e. impedance cardiography) and recognize the importance of modeling dynamic changes in physiology over time (Hastings & Kahle, 2019).

### Clinical implications

The current study points to the downstream effects of maternal ER difficulties early in development and suggests that maternal invalidation and child physiological reactivity interact to increase risk for aggression, particularly among at-risk youth. Findings highlight the significance of integrating these factors into etiological models of aggression and underscore potential treatment implications. For example, research demonstrates that targeting maternal ER improves parenting behaviors (Martin, Roos, Zalewski, & Cummins, 2017; Zalewski, Lewis, & Martin, 2018), and our own preliminary work in this sample has demonstrated that treatment-driven improvements in maternal ER difficulties has indirect effects on child externalizing symptoms 1 year later (Byrd, Lee, Frigoletto, Zalewski, & Stepp, 2021). Moreover, emerging work suggests that targeting maternal ER in combination with maternal validation training may further enhance child outcomes (Havighurst & Kehoe, 2017; Highlander et al., 2022), and our data suggest that maternal validation training for mothers with ER difficulties may be especially indicated when their children show heightened physiological reactivity to frustration. Moreover, given the extent to which intense negative emotion and aggression are intricately intertwined, strategies that help parents to separate the valid emotional experience from the maladaptive aggressive response may be useful. Alternatively, directly targeting children’s physiological reactivity to frustration (e.g. eliciting the dive reflex to activate the PNS; Rathus & Miller, 2000) may be especially effective in the context of maternal invalidation. Taken together, these findings suggest that targeted prevention and intervention efforts, prior to the emergence of severe psychopathology, may have the potential to shift trajectories of at-risk children.

## Supplementary Material

2023 Byrd et al Supp Mat

## Figures and Tables

**Table 1.** Descriptive statistics for all study variables by maternal ER difficulties

	Total sample (*n*= 77)			ER difficulties (*n*= 39)		No ER difficulties (*n*= 38)	
Demographic variables	Range	M/%	s.d.	M/%	s.d.	M/%	s.d.

Maternal characteristics							

Age (years)	22–45	33.17	4.83	32.38^a^	5.55	33.98^a^	3.87

Minoritized status	–	35%	–	51%^a^	–	18%^b^	–

Public assistance	–	38%	–	56%^a^	–	18%^b^	–

Child characteristics							

Age (months)	37–49	42.48	3.78	42.00^a^	4.05	42.97^a^	3.47

Sex (female)	–	56%	–	51%^a^	–	61%^a^	–

Minority status	–	47%	–	64%^a^	–	29%^b^	–

Primary study variables							

Maternal invalidation	1.31–4.61	2.39	0.67	2.69^a^	0.72	2.09^b^	0.46

RSA baseline	2.81–9.41	6.02	1.38	6.35^a^	1.34	5.68^b^	1.35

RSA frustration task	2.50–9.03	5.40	1.18	5.54^a^	1.13	5.27^a^	1.24

RSA reactivity	−2.58–0.64	−0.62	0.68	−0.81^a^	0.73	−0.42^b^	0.55

Aggression	0–36	7.18	8.58	10.23^a^	10.59	4.30^b^	4.73

Note. ER, emotion regulation; M, mean; RSA, respiratory sinus arrythmia; s.d., standard deviation. Means designated with different subscript letters are significantly different from each other (*p* < 0.05) based on post-hoc independent sample t tests.

**Table 2.** Bivariate correlations between primary study variables

	1	2	3	4	5	6	7	8	9	10
1. Maternal age (years)										
2. Maternal minority status	−0.13									
3. Receipt of public assistance	0.00	0.16								
4. Child age (months)	0.07	0.07	0.06							
5. Child sex (female)	0.20	0.11	−0.12	0.14						
6. Child minority status	−0.16	**0.78** **	0.19	0.01	0.15					
7. Maternal invalidation	−0.03	**0.36** **	0.18	0.00	−0.13	**0.36** **				
8. Child RSA baseline	−0.09	**0.24** *	0.16	0.01	−0.21	**0.36** **	0.11			
9. Child RSA frustration task	0.03	0.20	0.06	0.00	−0.07	**0.30** *	0.04	**0.87** **		
10. Child RSA reactivity	**0.23** *	−0.13	−0.21	−0.02	**0.29** *	−0.21	−0.16	**−0.51** **	−0.03	
11. Child Aggression	−0.11	0.18	**0.25** *	−0.08	−0.05	0.13	**0.34** **	0.00	−0.08	−0.14

*Note*. Significant effects are bolded.

* *p* < 0.05

** *p* < 0.01

## Footnotes

1 Ethnoracial composition for mothers was 65.6% White, 27.8% Black, 3.3% Hispanic/Latino, and 6.7% biracial multiracial.

2 Ethnoracial composition for children was 58.9% White, 25.6% Black, 5.6% Hispanic/Latino, and 15.6% biracial multiracial.

3 The COVID-19 global pandemic impacted our ability to complete in-person follow-up assessments. We were unable to complete any in-person assessments after March 2020 due to the nation-wide shutdown and research restrictions that limited prolonged in-person contact.

4 These two tasks were alternated across the four longitudinal assessments to mitigate practice effects associated with tasks including an element of deception (Monks, Smith, & Swettenham, 2005); thus, the baseline assessment included both tasks.

5 Physiological data were unavailable for four children. This included three children for whom physiological data were not available due to child refusal to wear electrodes and one child whose data were not usable due to staff error (i.e. incorrect placement of electrodes).

6 This included 59 teachers within a preschool/daycare facility, two in-home daycare providers, two alternative family members who cared for the child outside of the home, and one alternative family member who cared for the child inside the home. Missing data included seven children who were not yet enrolled in preschool/daycare, two children whose parent did not give consent to contact their teacher, one child whose teacher declined participation, and three children for whom data were determined to be invalid (e.g. teachers completed the questionnaire in less than 5 min). Participants with teacher data did not differ from those without any demographic variables, except for child age. Children with missing teacher data were younger (*t* = −2.34, *p* = 0.02).

7 Given debate about the degree to which RSA is truly a product of vagal activity v. an artifact of heart rate (HR; e.g. Monfredi et al., 2014), and recent research highlighting the potential non-trivial implications of examining RSA in isolation (i.e. without accounting for HR), we followed state of the art recommendations by de Geus, Gianaros, Brindle, Jennings, and Berntson (2019). Specifically, all models were re-run using: (1) HR instead of RSA; (2) HR as a covariate; and (3) RSA adjusted for HR. Regarding (1), there were no significant associations with primary predictors (maternal ER difficulties) or outcomes (aggression); regarding (2 and 3), results mirrored those reported above, suggesting that the present findings can be interpreted as effects of relatively unconfounded or largely vagally mediated influences on RSA.

† The notes appear after the main text.
