# Supplementary material for: Maternal invalidation and child RSA reactivity to frustration interact to predict teacher-reported aggression among at-risk preschoolers
Source: Psychol Med. Author manuscript; Available in PMC 2024 Oct 1. (PMC10520353; doi:10.1017/S0033291722003713)
Supplement: 2023 Byrd et al Supp Mat [file NIHMS1872470-supplement-2023_Byrd_et_al_Supp_Mat.docx]

**Supplementary Materials**

**Maternal invalidation and child RSA reactivity to frustration interact to predict teacher-reported aggression among at-risk preschoolers**

Byrd, A.L., Frigoletto, O.A., Vine, V., Vanwoerden, S., Jennings, R.D,

Zalewski, M. & Stepp, S.D.

**Table S1**. Prevalence of DSM-5 Diagnoses

| **Diagnosis** | **%** |
| --- | --- |
| **Any Personality Disorder** | **42%** |
| Borderline Personality Disorder | 36% |
| Avoidant Personality Disorder | 17% |
| Obsessive Compulsive Personality Disorder | 13% |
| Antisocial Personality Disorder | 9% |
| Paranoid Personality Disorder | 9% |
| Narcissistic Personality Disorder | 1% |
| **Any Mood Disorder** | **44%** |
| Major Depressive Disorder | 42% |
| Bipolar Disorder | 2% |
| **Any Anxiety Disorder** | **30%** |
| Generalized Anxiety Disorder | 22% |
| Panic Disorder | 10% |
| Social Anxiety Disorder | 9% |
| Specific Phobia | 7% |
| Obsessive Compulsive Disorder | 6% |
| Post-Traumatic Stress Disorder | 2% |
| Agoraphobia | 2% |
| **Any Substance Use Disorder** | **17%** |
| Substance Use Disorder | 11% |
| Alcohol Use Disorder | 10% |
| **Any Eating Disorder** | **11%** |
| Binge Eating Disorder | 10% |
| Bulimia | 1% |
| **ADHD** | **20%** |

*Note.* Diagnostic information was obtained from the Structured Clinical Interview for DSM-5 (SCID-5) for all disorders except personality disorders which were obtained from the Structured Interview for DSM-IV Personality (SIDP-IV).
